# The influence of medical testing on patients’ health: an overview from the gynecologists’ perspective

**DOI:** 10.1186/1472-6947-13-117

**Published:** 2013-10-10

**Authors:** Jolande Y Vis, Myra CB van Zwieten, Patrick MM Bossuyt, Karel GM Moons, Marcel GW Dijkgraaf, Kirsten J McCaffery, Ben Willem J Mol, Brent C Opmeer

**Affiliations:** 1 Department of Obstetrics and Gynecology, Academic Medical Center, Amsterdam, The Netherlands; 2 Department of General Practice, Academic Medical Center, Amsterdam, the Netherlands; 3 Department of Clinical Epidemiology, Biostatistics and Bioinformatics, Academic Medical Center, Amsterdam, The Netherlands; 4 Julius Center for Health Sciences and Primary Care, University Medical Center Utrecht, Utrecht, The Netherlands; 5 Clinical Research Unit, Academic Medical Center, Amsterdam, the Netherlands; 6 Screening and Test Evaluation Program, School of Public Health, University of Sydney, Sydney, Australia

**Keywords:** Test evaluation, Patient outcomes, Diagnostic test, Methodology, Qualitative research

## Abstract

**Background:**

A medical tests may influence the health of patients by guiding clinical decisions, such as treatment in case of a positive test result. However, a medical test can influence the health of patients through other mechanisms as well, like giving reassurance. To make a clinical recommendation about a medical test, we should be aware of the full range of effects of that test on patients. This requires an understanding of the range of effects that medical testing can have on patients. This study evaluates the mechanisms through which medical testing can influence patients’ health, other than the effect on clinical management, from a gynecologist’s perspective.

**Methods:**

A qualitative study in which explorative focus groups were conducted with gynecologists, gynecological residents and gynecological M.D. researchers (n = 43). Discussions were transcribed verbatim. Transcriptions were coded inductively and analyzed by three researchers.

**Results:**

All participants contributed various clinical examples in which medical testing had influenced patients’ health. Clinical examples illustrated that testing, in itself or in interaction with contextual factors, may provoke a wide range of effects on patients. Our data showed that testing can influence the doctor’s perceptions of the patients’ appraisal of their illness, their perceived control, or the doctor-patient relationship. This may lead to changes in psychological, behavioral, and/or medical outcomes, both favorably or unfavorably. The data were used to construct a conceptual framework of effects of medical testing on patients.

**Conclusions:**

Besides supporting clinical decision making, medical testing may have favorable or unfavorable effects on patients’ health though several mechanisms.

## Background

Medical therapies are often systematically evaluated for their clinical effects before they become common practice. On the other hand, medical tests are frequently implemented without a thorough evaluation of their actual influence on patients’ health. If medical tests or biomarkers are evaluated, it is most common to measure their ability to identify patients with the target disease or condition in terms of sensitivity and specificity. Yet, to make an evidenced based decision whether to include a medical test in clinical practice, one needs to assess all beneficial and harmful effects of using that test [1].

Medical tests may influence the health of patients by guiding clinical decisions, for instance if test positive patients are offered treatment. In addition, medical tests may also influence patients’ health through other mechanisms [1-4]. Testing may provide reassurance if patients feel uncertain about their condition [5,6] or provoke anxiety in case of bad news or indistinct test results [7]. By providing information to patients about their medical condition, tests may also trigger patients to alter their behavior and lifestyle [8].

In order to thoroughly assess the health-related consequences of medical tests, it is important to know how testing affects patients [1,9]. A clear understanding of the effects of medical tests on patients may help to optimize the benefits of these tests in clinical practice. At present, the variety of effects of medical testing on patients’ health and its mechanisms has not been systematically documented. Knowledge about all possible effects of medical tests on patients could be helpful for physicians to make optimal clinical use of medical testing in the widest sense of the term. This may include physical examinations, lab tests, tissue tests, imaging tests, invasive and non-invasive tests, and screening, diagnostic and monitoring tests.

This qualitative study explored the spectrum of effects of medical testing on patient outcomes from the gynecologists’ perspective, regardless of the clinical management decisions. These effects and their underlying mechanisms will be captured in a clarifying framework.

## Methods

We conducted a series of focus group discussions with medical practitioners. We involved medical practitioners rather than patients as they better oversee the function, impact and range of effects on patients of different medical tests for different medical conditions. The study was carried out in the field of obstetrics, gynecology, and reproductive health as this specialty covers a wide range of medical problems for a wide variety of patients.

### Sample

MD-PhD candidates working at gynecology departments, gynecological residents and house officers, and gynecologists working in peripheral or university hospitals were invited by personalized emails (purposive sampling by which we tried to obtain a wide variety of perspectives) [10]. The physicians signed up at their earliest convenience. Each focus group consisted of three to six physicians from the same hospital with similar functions. New focus groups were formed until enough data were available to answer the research question (data saturation). Since this study did only involve participation of physicians, not patients, our ethics committee (Medisch Ethische Toetsingscommissie AMC, Amsterdam, The Netherlands) did not require written informed consent. Nine focus groups were conducted in three different hospitals with in total 14 gynecologists, 19 residents (age 28–38) and 10 gynecological MD PhD students. All gynaecologists were 38 years or older with at least 10 years of clinical experience in gynecology, eight worked in an academic hospital at the time of the focus groups. All residents were 26–38 years had one to nine years of clinical work experience in gynaecology, one third had only worked peripheral hospitals, one third had only worked in university hospitals, and one third had worked both in peripheral and university hospitals. The MD-PhD candidates were 31 years or younger and had less than five years of clinical work experience, half of them in peripheral hospitals and half of them in university hospitals.

### Procedure

**Figure 1:**
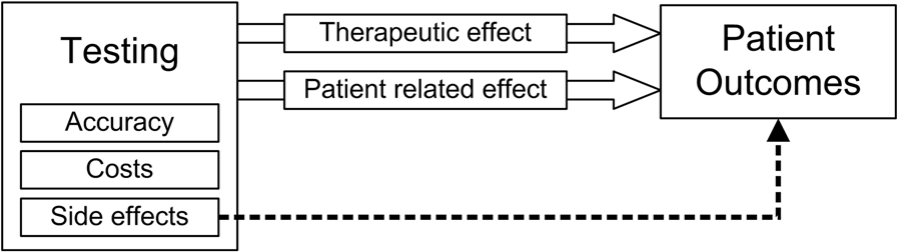
Effects of medical testing on patients outcomes.

The focus groups interviews were facilitated by MvZ (qualitative health researcher and ethicist), BO (clinical epidemiologist specialized in diagnostic test evaluation) or JV (medical doctor) in turn. Participants were briefly introduced to the background and aims of this study and asked to reflect on the following question: ‘Have you ever performed medical tests or investigations that may have influenced the health of your patients, regardless of the effects of subsequent clinical decisions?’ Medical tests were defined in the the widest sense of the term, including screening, prognostic, diagnostic, and monitoring tests by physical examinations, laboratory tests, tissue tests, imaging tests, invasive and non-invasive procedures, questionnaires and function tests. During the group discussions a figure illustrating our hypothesis of additional patient related effects of medical testing was displayed [Figure 1]. Group discussions were stimulated in an exploratory manner without any further prompt. All clinical examples were shared without being traceable to a specific person and thus respectful toward the privacy and rights of all patients. The duration of the focus group meetings took between 1 to 1.5 hours.

### Analysis

All focus groups were audio recorded and transcribed verbatim. For the purpose of this exploratory study, saturation of data was reached after nine focus groups, as no new information was gained in the last two focus groups. Sequential analyses according to the grounded theory method – a method by which new theories are generated from systematic data collection – were performed by MvZ, BO and JV to reduce individual biases [11]. Consensus about the analyses was reached through discussion amongst the whole research team.

After four focus groups, the transcripts of the focus group discussions were summarized and coded inductively, by giving each piece of (potential) relevant information a specific code that would fit that content best. Similar codes were grouped into broad concepts. These were discussed with an expert panel consisting of senior epidemiologists and a gynecologist (other authors). In this expert meeting we decided not to put emphasis on the medical consequences of the accuracy of a test (including false negative of false positive results), but to focus on the consequences of test information in general.

After six focus groups, JV, BO and MvZ independently performed a second interim analysis. Through successive data mapping and discussions, a preliminary coding frame of recurrent themes and concepts was constructed in consensus amongst the whole research team. We decided to use the input-throughput-output framework for further analysis, as this model fitted the different themes. Subsequently, MAXQDA2, a software program for qualitative data analysis, was used for systematic organization and storage of the data.

Data from all subsequent focus groups were coded deductively (with prespecified codes) according to the updated code tree. Recurrent themes were checked for group to group validation (a theme had to be recognized by at least two focus groups) and contradictive data were sought. New themes were sought and definitions were accentuated. During this iterative process, we structured all themes and concepts from the data in a conceptual framework, reflecting the mechanisms of effects of medical testing on patients.

## Results

All participants put forward a variety of clinical examples from their own practice that illustrated effects of medical testing on patients. The effects reported throughout the different focus groups were comparable. Most clinical examples consisted of three main elements. The first element describes factors within the particular context of a test situation that are essential for the course of such effects. The second element describes how testing or disclosing the test results can influence patients’ perception of their health or clinical situation. The final element encompasses subsequent effects on patient outcomes. For example, if testing changes patients’ health perceptions, this may in turn influence their emotional state and/or behavior.

*“Patients presenting with abdominal pain, they often have all kind of ideas about cancer or severe infection [contextual factors]. If you perform some tests, and you can tell them that the ultrasound looks good (…) Then the people are often reassured and think that it’s probably just some general intestinal pain, and that their abdomen will be ok again [perception]. Yes, I often see that some quick tests can reassure patients and that it decreases the severity of their complaints [patients’ outcomes].”* [Focus group 6]

We arranged these three main elements using the framework of an input-throughput-output model. In this framework, the contextual factors are considered to be the necessary input for the development of effects of medical testing on patients. The subsequent changes in patients’ perception are considered to be the throughput that leads to these effects. The output of the model consists of the effects on patients of medical testing themselves. This structure reflects the basic outline of the conceptual framework. The themes derived from the data could be fitted within this frame.

In the next paragraphs, we shall describe these themes in more detail, and then present the populated conceptual framework. We will thereby report a selection of factors within each theme, for illustration, rather than for being all-inclusive.

### A. Input: contextual factors

In the clinical examples, different contextual factors were described that could provoke or contribute to effects of testing on patients. Notably, the development of these effects did not only seem to depend on the medical test and the test result. It was also said to be influenced by the prior knowledge and beliefs of patients about their health and the characteristics of the clinical encounter.

#### ***A-1. Prior beliefs***

Effects of testing on patients were often seen to depend on the prior (health) beliefs of an individual patient. For instance, it was mentioned that persons with different personalities or anxiety levels may experience different effects of testing, even though they receive the same test result. Different assumptions about the cause of the symptoms, especially in patients with different medical histories, were often said to be an important factor [Table 1, citation 1]. Cultural differences and medical traditions were also frequently mentioned. For example, if patients are used to periodic check-ups, like a yearly cervical smear, they might feel uncomfortable without such annual reassurance [citation 2].

**Table 1:** A selection of citations from the focus group meetings

	**Input: contextual factors**
1	*“It is a completely different story if a woman had a previous stillbirth or if the woman is happily pregnant and seeing a midwife.”* (FG 1)
2	*“If we have frightened patients, like DES-daughters with increased risk for clear cell carcinoma, it is hard to convince them that they are not at risk anymore at a certain age.”* (FG 3)
3	*“You have the risk of false positive test results (…) you know that causes anxiety.”* (FG 3)
4	*“An image is much more illustrative for patients. (…) it is nicer for patients to see it themselves directly than to interpret a lab result, how would they know what a normal result is?”* (FG 4)
5	*“You can say either ‘I don’t see anything wrong’ or you can say ‘everything looks absolutely marvelous’. It is all subjective; it depends on how you wrap the message.”* (FG 2)
6	*“That woman goes through a rough time, and I am not willing to talk about it for 30 minutes each week. But she will be able to talk to a nurse during a CTG (…) and she will be happy about the good results.”* (FG 1)
	**Throughput: perception**
7	*“If a patients feels fatigued but the hemoglobin is normal, it illustrates that there must be another cause for their fatigue than anemia.”* (FG 7).
8	*“This woman received palliative treatment for metastasized ovarian cancer. But she used tumor markers as a measurement of her health, irrespectively of whether she felt healthy or sick.”* (FG 3).
9	*“By giving a diagnosis, you show patients that they are not to blame for developing the illness.”* (FG 4)
10	*“I think many patients want a label for their disease, to help them feel in control again. (…) that can really help a patient.”* (FG 5).
11	*“I do the test to convince the patient that her vaginal discharge is normal, she wouldn’t accept it as being normal without a test (…) then she wouldn’t have felt taken seriously.”* (FG 9)
12	*“The chance that a patient will go to a different hospital increases if we withhold from any medical test.”* (FG 1)
	**Output: patient outcomes**
13	*“A test that can give much reassurance is the cervical length measurement. (…) Patients attach a large amount of importance to that result, so if you give them the length measurement and it is large enough, they are okay again for a while.”* (FG 8)
14	*“I have a patient who is finally diagnosed with endometriosis. This diagnosis alone can give a bit of peace and helps to cope with the complaints, because now the patient knows what causes the complaints for a fact, and that gives her comfort.”* (FG 7)
15	*“You have to be careful in performing tests for reassurance, this can undermine patients’ confidence that things will turn out all right by themselves. Then they might need a reassuring test for every little symptom!”* (FG 5)
16	*“A glucose control can be very confronting for a woman with diabetes; they might skip the next piece of cake.”* (FG 6)
17	*“Even if you find no impairment caused by smoking, it might still kill her if she continues. But her smoking behavior can be strengthened by the test, because she might reason that the test result shows that she is immune for the negative effects of smoking.”* (FG 7)
18	*“For example, if a woman has anemia, but she dislikes iron tablets, then you can order a hemoglobin test to motivate her to continue the tablets. You can say that her hemoglobin value has improved, but has not yet recovered…”* (FG 4)
19	*“If you can reassure people about certain things, some people perceive less pain or have fewer complaints. It is a sort of homeopathic principle.”* (FG 3)
20	*“Even a normal cyst can make people ill (…) they will develop abdominal pain immediately.”* (FG 7)

FG = focus group.

#### ***A-2. Medical test***

Some effects of medical tests on patients were reported to be a consequence of the test characteristics or the test results. Physicians were often concerned about false positive test results, which can induce unnecessary anxiety in healthy patients, and also inconclusive results that can neither confirm nor reject a certain condition and provoke anxiety [citation 3]. Overall participants thought that imaging tests more frequently generated effects on patients as compared to non-imaging tests, in both positive and negative ways. As imaging tests show patients a visual image of their body, it was said that these tests results can be more illustrative and convincing to patients [citation 4]. Imaging tests were also often mentioned for their unintended incidental findings, which may be spurious but can cause major anxiety in patients.

#### ***A-3. Clinical encounter***

We found a profound role for the clinical encounter in generating effects of testing on patients. The way of communicating medical information and the test result was said to be important. Emphasizing favorable results both verbally and nonverbally was thought to contribute substantially to patient effects [citation 5]. Alternatively, tests were sometimes used instrumentally, as part of the clinical encounter. By performing a test, the physicians could give medical attention to patients to enhance their satisfaction about the clinical encounter [citation 6].

### B. Throughput: perception

In almost all clinical examples testing had altered patients’ perceptions of their health. We found three recurrent mechanisms by which testing could affect health perceptions in patients. The most frequently mentioned mechanisms were a change in patients’ appraisal of their medical situation and in patients’ perceived control over their situation. Testing was also said to the influence of testing on the doctor-patient relationship.

#### ***B-1. Appraisal of illness***

An important function of testing was seen in improving patients’ understanding of their disorder. Tests were said to contribute to patients’ comprehension of their medical condition or the cause of their complaints. This effect was described either when the presence of disease was reflected or if absence of disease was shown [citation 7]. It was noticed that a test result was often perceived by patients as an objective sign of their complaints or health status, even if it reflected subclinical changes [citation 8]. More general, knowledge about their condition was thought to help patients in better understanding and anticipating on the likely course of their disease.

#### ***B-2. Perceived control***

Another mechanism by which testing was seen to affect patient outcomes was by influencing the perceived level of control patients felt they had over their health. This was noticed, for instance, that patients sometimes felt guilty or troubled in situations where they experienced unexplained symptoms or complaints. Diagnosing an acknowledged disease was seen to help ease their guilt [citation 9]. It was also said that most patients with unexplained complaints were searching for a defined label for their symptoms, to have something to go by [citation 10]. Tests were also used for increasing adherence, by showing that patients can have some control over their disease. For instance, tests that evaluate an intervention may illustrate patients that adherence to a certain treatment or lifestyle is effective for them.

#### ***B-3. Doctor - patient relationship***

Many participants used medical testing to convince patients of the value of the proposed management strategy, especially if patients did not initially trust their health care providers’ opinion. For example, as some patients did better trust a diagnosis if it is grounded on objective parameters, physicians were not always able to convince patients that an extra medical test would not be beneficial [citation 11]. Physicians also reported that denying a patient a medical test may make patient feel that they are not taken seriously and that they are getting sub-standard healthcare. This was seen as having the potential to lower patients’ adherence to medical advice [citation 12]. Study participants also said that more extensive counselling could not always replace the comforting effects of a medical test. Testing was sometimes necessary to maintain patient satisfaction and the perceived quality of care.

### C. Output: patient outcomes

Several effects of medical testing on patient outcomes were recognized in the clinical examples. We distinguished three main categories: psychological outcomes, behavioral outcomes and medical outcomes.

#### ***C-1. Psychological outcomes***

It was reported that the testing procedure itself could influence the emotional state of patients, regardless of the test result. For instance, testing and waiting for results could be stressful for patients, this could lead to anxiety. Yet, more often, reassuring effects of tests were mentioned [citation 13]. Providing patients with a clear diagnosis was also believed to have positive effects on patients, as they may be more willing to accept their medical situation and get recognition for their complaints [citation 14]. On the other hand, some patients were said to develop a dependence on reassuring test results to keep a healthy feeling. In such cases, patients could get very concerned about their health without repeated instrumental reassurance [citation 15]. This negative effect was reported for a wide range of screening, diagnostic and prognostic tests.

#### ***C-2. Behavioral outcomes***

It was also reported that medical testing can affect patients’ behavior. Physicians sometimes ordered tests to confront patients with the necessity to change their behavior [citation 16]. It was questioned whether such confronting tests would always have beneficial effects on the behavior of patients, as good test results might reinforce unfavorable behavior [citation 17]. Additionally, focus group participants emphasized patients’ outcomes in terms of adherence. Improving patients’ understanding of their condition and reinforcing the doctor-patient relationship through testing was said to stimulate adherence. Alternatively, it was reported that evaluating the therapeutic effects could encourage patients to continue with their therapy [citation 18].

#### ***C-3. Medical outcomes***

Participants mentioned that psychological effects of testing may also influence medical outcomes. It was said that reassurance by negative tests results may lead to a reduction in complaints [citation 19]. Receiving a positive test result was sometimes seen to increase the level of complaints, as this was thought to foster patients’ perceived illness [citation 20]. In addition, examples illustrated that effects of testing on adherence and lifestyle behavior may affect the course of the disease.

### D. Conceptual framework

**Figure 2:**
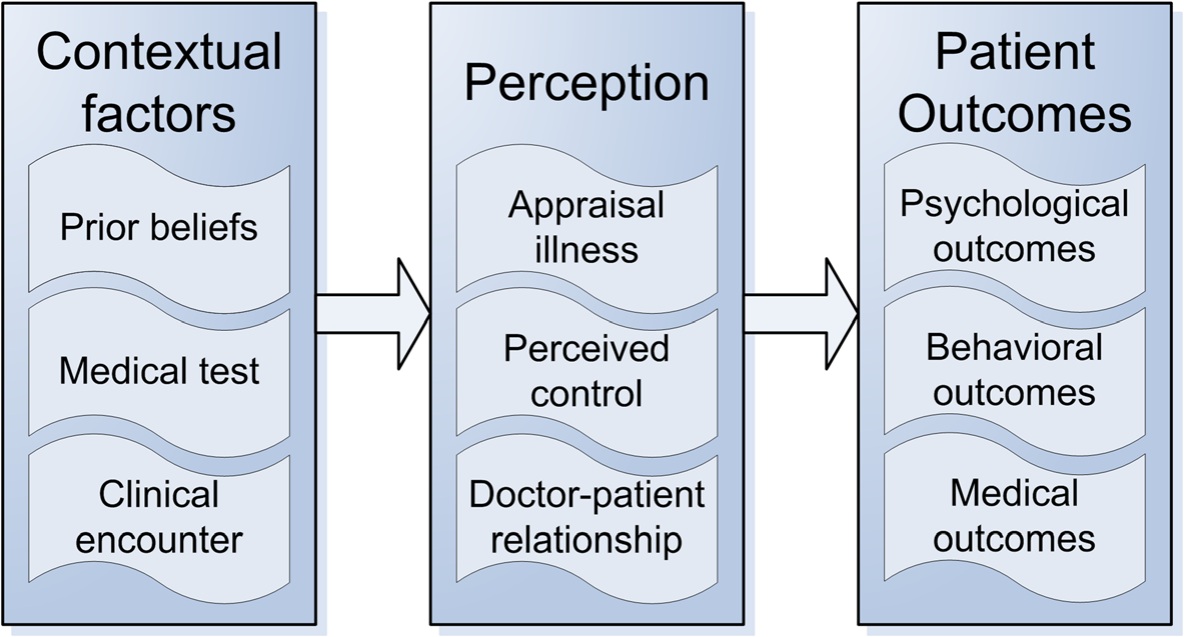
Conceptual framework of effect of medical testing on patients.

Throughout the clinical examples it became apparent that different contextual factors around medical testing (input) could trigger changes in patients’ perception (throughput), which in turn could result in additional effects on health outcomes (output). Based on our qualitative data analysis and synthesis, we arranged the mechanisms and outcomes in an overall model that reflects the effects of medical testing on patients. This conceptual framework is summarized in Figure 2. As our analyses were qualitative and descriptive, we have outlined general patterns of mechanisms and effects of testing on patients, without defining all specific relationships between different factors.

## Discussion

This study evaluated whether and how medical testing can influence patients’ health through other mechanisms than clinical management. Our results confirmed that health care professionals recognize the presence of a wide range of potential effects of testing on patients, which operates through several mechanisms. These effects of testing may favorably or unfavorably affect patient outcomes in clinical practice. For instance, it was reported that patients place great value on the reassurance gained from a negative test result from a feared outcome or, in case of a positive test result, having a medical explanation for symptoms even when there is no significant management change. Our study demonstrated the importance of individual circumstances and the clinical encounter in the appraisal of medical tests [5,12].

To our knowledge, this is the first study to systematically explore effects of medical testing on patients from a clinicians’ perspective. Many mechanisms for generating these effects of testing on patients have been previously described in adjacent disciplines, such as health psychology and behavioral medicine. For example, the effects of testing on anxiety and reassurance of patients are widely acknowledged [3,6,13]. The influence of health beliefs and the doctor-patient relationship on medical adherence has also previously been examined [14]. Yet, a systematic assessment of such additional effects of testing on patients is usually not addressed or considered in medical evaluations of medical tests [15,16]. We suggest that these effects are incorporated in future test evaluations if these effects are considered to be relevant for patients, though it remains a challenge to measure and weigh the value of these effects against more traditional outcomes.

In some focus groups, the reasons for ordering tests were also discussed. Many physicians acknowledged that they regularly ordered tests with the intention to provoke an effect on their patients. Tests were sometimes ordered to stimulate adherence, to elicit reassurance, or in response to patients’ preferences in order to maintain a good physician-patient relationship [17]. We observed that physicians’ reported primary reasons for ordering a test were not always in line with the effects of that test. For example, whereas physicians may focus on detecting fetal abnormalities to give couples an opportunity to abort the pregnancy in case of malformations, future parents often only want to be reassured and informed about the gender. Yet, several focus groups pointed out that an increased usage of medical tests might also cause dependence of these tests for achieving reassurance and a healthy feeling. An increased availability of medical tests could increase the future demand for such tests.

Although outside the primary focus of our study, focus group discussions addressed non-health related consequences of tests as well, such as financial planning (concerning mortgage, insurance and career development) and consequences for relatives or family planning. We did not include these other consequences in our analyses, as we restricted our focus to health-related effects on patients, although such consequences are increasingly assessed in studies evaluating different forms of genetic testing [18-20].

A limitation of this study is that the results are only based on discussions with health care providers in obstetrics, gynecology and reproductive health. It reflects their perspective on the role and effects of testing on patients, mostly females. Although this medical specialty encompasses a broad spectrum of care, including oncology, elective, acute, chronic, outpatient and inpatient care, further research is needed to confirm that our results also apply to other medical areas. It is also possible that some effects medical tests differ between genders. In addition, social and cultural context of patients and physicians may affect the occurrence or magnitude of these effects. Different types of test (e.g. invasive versus non-invasive, histology versus biomarker etc.) could also elicit different effects. Unfortunately, our data provides insufficient information to further explore this item as we did not intend to distinguish effects between different types of medical tests throughout the focus groups. Whether the range of effects of medical testing would be similar when reported by patients themselves is currently being investigated.

It remains a challenge to define how the effects identified in this study can be more systematically addressed in future test evaluations. In our view, researchers and decision-makers have to be aware that testing can generate beneficial or harmful effects on patients regardless of subsequent clinical decision making. Researchers need to recognize the relevance of such effects in specific testing situations. For this purpose, it could be useful to develop a tool that assists in identifying effects of medical testing on patients. Subsequently, health researchers will need instruments to quantitatively measure and summarize the effects. Researchers and decision-makers have to work together in developing ways for incorporating these effects in comprehensive test evaluations and for weighing them against more conventional clinical outcomes. In the end, more comprehensive test evaluations that incorporate the full range of effects of testing on patients will lead to better decisions about testing and, consequently, to a more effective and efficient health care system.

## Conclusion

Testing may affect the perceptions of patients depending on their prior beliefs, the properties of the medical test, and the clinical encounter. This may change patients’ perception of the appraisal of their illness, perceived control, or doctor patient relationship. This may in turn influence patients’ health through psychological outcomes, behavioral outcomes, or medical outcomes. We suggest that both clinicians and health researchers take these potential additional effects of medical testing on patients into account within their test recommendations.

## Competing interests

All decisions concerning the study design, execution, analyses and reports were made solely by the investigators. The authors declare that they have no competing interests.

## Authors’ contributions

JV, MvZ, and BO conducted the focus group interviews and performed the primary analyses. PB, KM, MD, KMC and BWM participated in the coordination of the study. All authors participated in the design of this study. JV drafted the first version of the manuscript. All authors helped to finalize the manuscript and approved the final version.

## Pre-publication history

The pre-publication history for this paper can be accessed here:

http://www.biomedcentral.com/1472-6947/13/117/prepub
